# A Contactless and Biocompatible Approach for 3D Active Microrobotic Targeted Drug Delivery

**DOI:** 10.3390/mi10080504

**Published:** 2019-07-31

**Authors:** Federico Ongaro, Dennis Niehoff, Sumit Mohanty, Sarthak Misra

**Affiliations:** 1 Surgical Robotics Laboratory, Departmen of Biomechanical Engineering, University of Twente, 7522 NB Enschede, The Netherlands; 2 Surgical Robotics Laboratory, Department of Biomedical Engineering, University Medical Centre Groningen, University of Groningen, 9713 AV Groningen, The Netherlands

**Keywords:** microrobotics, surgical robotics, minimally-invasive surgery, ultrasound tracking, magnetic actuation, targeted drug delivery

## Abstract

As robotic tools are becoming a fundamental part of present day surgical interventions, microrobotic surgery is steadily approaching clinically-relevant scenarios. In particular, minimally invasive microrobotic targeted drug deliveries are reaching the grasp of the current state-of-the-art technology. However, clinically-relevant issues, such as lack of biocompatibility and dexterity, complicate the clinical application of the results obtained in controlled environments. Consequently, in this work we present a proof-of-concept fully contactless and biocompatible approach for active targeted delivery of a drug-model. In order to achieve full biocompatiblity and contacless actuation, magnetic fields are used for motion control, ultrasound is used for imaging, and induction heating is used for active drug-model release. The presented system is validated in a three-dimensional phantom of human vessels, performing ten trials that mimic targeted drug delivery using a drug-coated microrobot. The system is capable of closed-loop motion control with average velocity and positioning error of 0.3 mm/s and 0.4 mm, respectively. Overall, our findings suggest that the presented approach could augment the current capabilities of microrobotic tools, helping the development of clinically-relevant approaches for active in-vivo targeted drug delivery.

## 1. Introduction

Considered a figment of imagination until a few decades ago, microbotic surgeries are steadily approaching clinically-relevant scenarios [1,2]. Over the past years technological advances in the fields of nanofabrication and microrobotic control have allowed to miniaturize and control robots as small as a few microns [3,4]. Consequently, microrobotics has been drawing significant attention for minimally invasive surgeries, such as biopsies, cytoreductions and endarterectomies, as well as cardiac and ophthalmic surgeries [5,6].

Among these interventions, microrobotic targeted drug delivery is arguably the closest to the reach of the current technology [7,8]. In point of fact, techniques have been presented to miniaturize microrobots well beyond the constraints of the main human vascular vessels [9]. Moreover, microrobots have been demonstrated to be capable of navigation in three dimensional (3D) and dynamic environs [10,11]. Therefore, drug-coated microrobots, capable of navigating in unpredictable environments, would be able to perform targeted drug delivery inside the human body. Not only would this microrobotic approach supersede the current approach in terms of effectiveness of drug delivery, but also under numerous other aspects, such as reduction of side effects as well as improved regulation of drug-intake [8].

However, most microrobotic research is still mainly confined to controlled environments [9,10,11]. In fact, the untethered and biocompatiblity requirements pose a main challenge to the development of sensors and actuators for in-vivo microrobotic testing. Particularly, a clinically-relevant microrobotic system has to possess biocompatible propulsion mechanisms (1), imaging systems (2), and it also has to be able to execute tasks beyond those required for navigation (3).

The development of biocompatible microrobotic propulsion mechanisms (1) is challenging due to scale requirements. Customary battery-powered actuators cannot be used, as the current technology does not allow sufficient miniaturization of on-board power sources. Consequently, researchers have investigated the use of chemical, thermal, acoustic, and electromagnetic microactuators capable of wirelessly harnessing power stored within the material of the microrobot or its surroundings [12,13,14]. Nonetheless, due to power attenuation and lack of biocompatibility, a considerable number of these actuators is either incompatible or only compatible with a limited number of clinical procedures [9].

Also imaging techniques present a significant hurdle with regard to clinical compatibility (2). Historically, optical images have been used to sense the state of microrobots. Yet optical cameras are not suitable for minimally invasive clinical interventions, as they would require incisions for their insertion. However, biocompatible alternatives often suffer from major drawbacks, such as limited bandwidth, low resolution, artifacts or long-term toxicity. Therefore, robust tracking procedures have to be developed for pose reconstruction.

Finally, to perform minimally invasive surgery, microrobots generally have to be capable to execute actions beyond those required for navigation (3). Case in point, active drug-delivery microrobots need to perform chemical release of substances. Clearly, also the actuators for such actions have to be untethered and biocompatible, as they must not unintentionally alter physiological parameters, such as pH, temperature, or intravascular pressure.

In this study, we present a fully biocompatible approach for microrobotic targeted drug delivery that addresses these challenges. Such approach exploits the physical properties of the used microrobot to develop a contactless actuation and sensing system that would only affect the targeted tissue. In particular, medical ultrasound imaging is used for position sensing, while quasi-static and high-frequency magnetic fields are used for navigation and active drug release, respectively. This approach is then validated using an anatomically accurate phantom of the human vascular network. Overall, this proof-of-concept work presents the first closed-loop active targeted drug-model delivery using a thermoresponsive coating and a fully biocompatible microrobotic system. Moreover, our results show reductions of several orders of magnitude in completion time of the task with respect to previous literature using similar technology [15].

## 2. Materials and Methods

**Figure 1:**
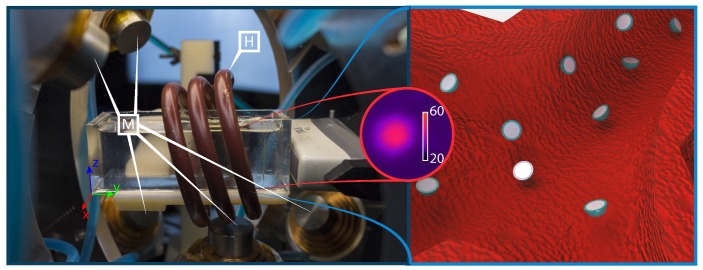
(**Left**) A close-up image of the used setup depicting the ultrasound probe, as well as the coils used to generate high frequency $\left(\begin{array}{c} H \end{array}\right)$ and quasi-static magnetic fields $\left(\begin{array}{c} M \end{array}\right)$. (**Central inset**) One of the used microrobots after 15 s of induction heating (18 mT at 126 kHz), captured using a cooled-detector thermal camera with 12 mm extension ring (FLIR Systems, Wilsonville, OR, USA). Scalebar in degree Celsius. (**Right**) Illustration of (sectioned) proposed microrobots navigating a blood vessel to perform targeted drug delivery.

This section presents the custom setup and the techniques used to perform clinically-relevant targeted delivery of a drug-model. From a hardware perspective an electromagnetic testbed, a generator of high-frequency magnetic fields, a clinical ultrasound machine, and an anatomically accurate phantom are used (Figure 1) [16]. Moreover, we develop software algorithms for the control of the electromagnetic testbed, as well as for control and tracking of the microrobots.

### 2.1. Electromagnetic Setup

**Figure 2:**
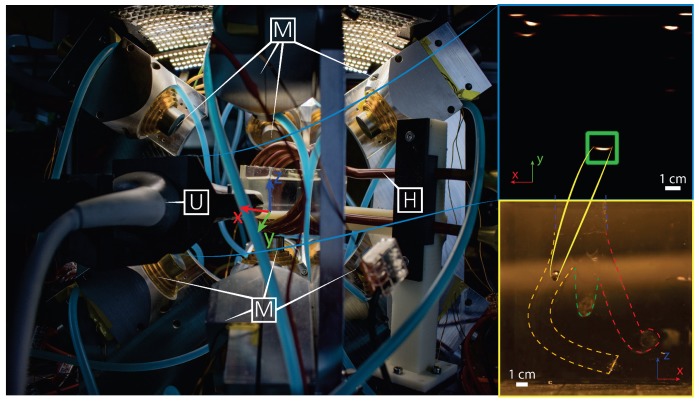
The electromagnetic setup. The nine metal-core electromagnets $\left(\begin{array}{c} M \end{array}\right)$ are capable of generating quasi-static fields of up to 10 mT while allowing access to objects smaller than a φ160 mm sphere. A linear stage is used to move the ultrasound probe $\left(\begin{array}{c} U \end{array}\right)$ along the gravitational axis (z). Finally, the liquid-cooled high frequency coil $\left(\begin{array}{c} H \end{array}\right)$ can be seen in the right end of the image. (**Top-right inset**) A representative B-mode ultrasound image. The position of the microrobot is marked by a green square. Due to diffraction, artifacts, and noise the footprint of microrobot in the ultrasound image is significantly larger than its real size. (**Bottom-right inset**) A coated microrobot navigating the phantom of vascular vessels (outlined by the colored dashed lines).

The electromagnetic setup used for motion control of the microrobots consists of nine metal-core electromagnets and a camera attached to a microscope (Figure 2). While the setup was originally presented in our previous work, several modifications have been done to address for this study [10]. Most notably, a coil (Ultraflex Power Technologies, New York, USA) for the generation of high-frequency magnetic fields has been added to the setup (Figure 1). Additionally, a linear stage (MISUMI, Tokyo, Japan) is now used to move the ultrasound probe (SIEMENS AG, Erlangen, Germany) in directions orthogonal to the imaging plane (Figure 2). The addition of this linear stage allows to use a traditional 2D medical ultrasound for 3D tracking of the microrobots. Conversely, the additional coil is used to generate sinusoidal electromagnetic fields used to trigger the release of the drug-model.

### 2.2. Ultrasound Tracking

High-frequency acoustic waves are used for biocompatible tracking. For this purpose, an 18 MHz transducer is connected to a 2D medical ultrasound machine (SIEMENS AG, Erlangen, Germany). As the used transducer only allows two-dimensional imaging, additional procedures are required to obtain the 3D position of the microrobot. Specifically, the additional component has to be detected by allowing the ultrasound probe to move orthogonally to the image plane.

An intuitive approach would be to use this motion to follow the microrobot in the workspace, maintaining it always in the image plane. However, due to the presence of noise and artifacts, changes in the imaged size of the robot do not reflect the actual footprint of the scanned section. Consequently, it is challenging to compute the gradient of the position of the microrobot, which is required to follow its movements with the ultrasound probe.

**Figure 3:**
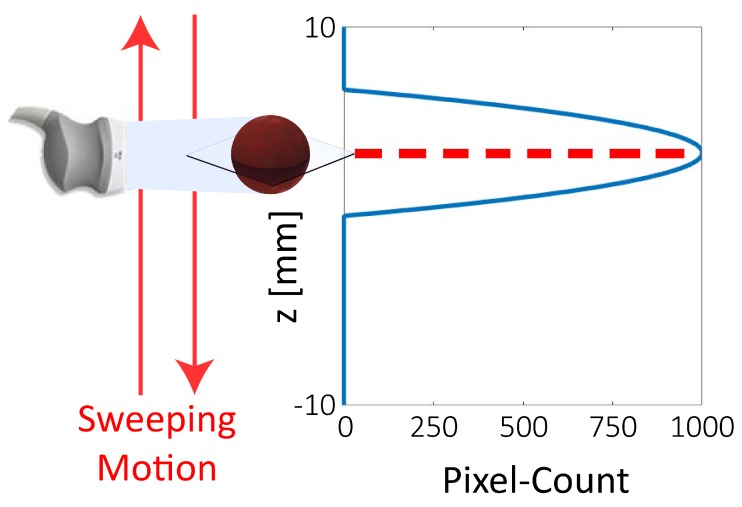
Schematic depicting the approach to determine the out-of-plane component (*z*) in the tracking procedure. The ultrasound transducer continuously sweeps the workspace at a frequency of 1 Hz, hence providing two positions per second. The in-plane (*x* and *y*) components of the position of the microrobot are tracked using the procedure illustrated in Figure 4. The graph shows the dependence of the tracked blob size (blue line) on the position of the transducer. This dependence is used to triangulate the position of the microrobot using the point of maximum size (marked by the dashed red line).

**Figure 4:**
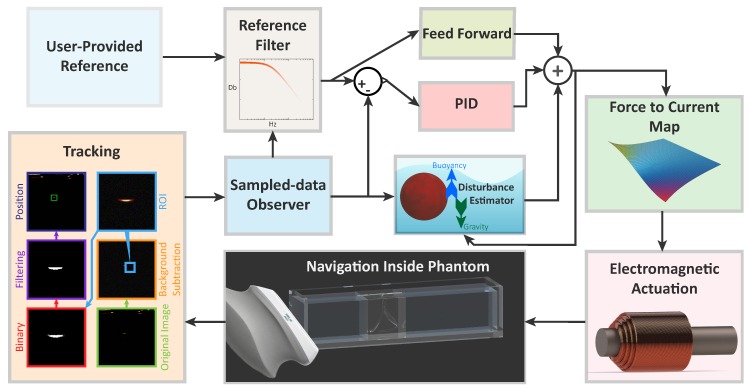
Schematic of the control loop. The user provides the position reference. This is preprocessed by a fourth order filter that removes frequency components above the bandwidth of the controller and ensures continuous derivatives. This prevents the filtered reference from increasing with a dynamic that is faster than the maximum one of the controller The filtered reference is then provided to a Proportional, Integral and Derivative (PID) controller. This controller designed to minimize disturbances with frequencies higher than a decade below that of the tracker [17]. A feedforward component is added to improve the control performance. As the low-level controllers feed the currents determined by the force-to-current map, the microrobot moves. This motion is detected by the ultrasound tracking algorithm using the procedure shown in the image (combined with the approach of Figure 3). Such tracking procedure begins using a Gaussian mixture-based segmentation algorithm for background subtraction [18]. A Region Of Interest (ROI) around the estimated position of the microrobot is then selected and binarized using a variable-threshold. A dilation morphological filter is then applied to the image. Finally, the center of the largest blob is selected as the position of the microrobot. The computed position is then provided to a sampled-data observer, which provides the controller with intersample state estimations [19].

Alternatively, we use a sonar-inspired approach. In this approach the ultrasound transducer is swept along the height of the workspace, while a Region Of Interest (ROI) in the surrounding of the estimated position of the microrobot is scanned for 2D detection (Figure 3 and Figure 4). The joint variable of the stage in the point of detection, can then be used to triangulate the position of the microrobot. While this approach results in a reduced bandwidth of the tracking algorithm, it also provides a significant gain in robustness with respect to a gradient-based approach. Overall, this approach grants extremely robust tracking at a frequency of 2 Hz.

### 2.3. Microrobot Selection

The microrobots used in the study are selected in order to satisfy a set of conditions. First, the microrobots must have a continuous and smooth surface to guarantee uniform drug release, as well as to minimize contact pressures on the vessels in case of collisions. Second, the microrobots size must be small enough to grant access to the major body vessels, while remaining sufficiently bigger than the ultrasound resolution (100 μm) to minimize artifacts. Third, the robot has to be able to withstand unaltered the temperatures required for drug release. Finally, we want the magnetic dipole moment of the microrobot to be as strong as possible. This allows to control the robot with weak magnetic fields and gradients, as the ones generated by distant electromagnets, effectively enlarging the accessible workspace. Addressing all these requirements we select Neodymium Iron Boron (NdFeB) microspheres with diameter of 800 μm for our study. In particular, we use N45 grade NdFeB, which has a Curie temperature of 80${}^{\circ }$ C and offers a remnant magnetization of 1.35 T. Moreover, NdFeB has a conductivity of 6.7×10${}^{6}$ S/m which renders it particularly sensitive to induction heating.

### 2.4. Induction Heating

In point of fact, microrobots are heated using high-frequency magnetic fields, exploiting the phenomenon commonly known as induction. Therefore, the heat generated in the microrobot is a result of both hysteresis losses and eddy currents [20]. However, due to the low permeability—at the magnetic fields reported in this study—of the pre-magnetized NdFeB microrobots, the heat generated due to hysteresis losses is minimal with respect to eddy losses. Consequently, a custom-coil is designed to maximize these effects (Ultraflex Power Technologies, New York, USA).

The resulting RLC circuit is capable of locking at two frequencies (126 kHz and 228 kHz) producing a field of 18 mT in amplitude. Depending on the magnetic energy stored in the electromagnetic field induced in the material, a power of up to 1.7 kW is required to generate such high-frequency fields. Part of such power is dissipated on the microrobot, while most of the remaining power is dissipated on the coil. In order to prevent overheating, such coil is designed to be hollow. This allows to run water inside the coil for cooling purposes (6.8 L/min at 3.4 bar). Finally, it is interesting to notice that the human body is not affected by these magnetic fields as it does not contain sufficient amounts of conductive or hard magnetic materials [16].

### 2.5. Motion Modeling and Control

After developing a testbed for drug release, we look at closed-loop motion control. For this purpose, we model the microrobots according to the following state space model:

(1) $$\left[\begin{array}{c} \dot{p_{i}}\\ \ddot{p_{i}} \end{array}\right]=\left[\begin{array}{cc} 0 & 1\\ 0 & -\frac{\rho C_{D}A}{2M} \end{array}\right]\left[\begin{array}{c} p_{i}\\ \dot{p_{i}} \end{array}\right]+\left[\begin{array}{c} 0\\ -\frac{1}{M} \end{array}\right]F_{em,i}+\left[\begin{array}{c} 0\\ -\frac{1}{M} \end{array}\right]d_{i},$$

where ρ∈R is the density of the medium, and $C_{D}\in R$, A∈R and M∈R are the drag coefficient, cross sectional area, and mass of the sphere, respectively (Table 1) [10]. In turn, $d\in R^{3}$ collects all the modeled components that are not influenced by the state of the system or by the control inputs. Moreover, $p_{i}\in R$, $F_{em,i}\in R$, and $d_{i}\in R$ are the *i*-th component of the position ($p\in R^{3}$), electromagnetic force ($F_{em}\in R^{3}$), and d, respectively. These are defined as follows:

(2) $$\begin{array}{c} p={\left[\begin{array}{ccc} x & y & z \end{array}\right]}^{T}, \end{array}$$

(3) $$\begin{array}{c} F_{em}=\nabla \left(B\cdot m\right), \end{array}$$

(4) $$\begin{array}{c} d=\Delta F_{d}+g\left(M-V\rho \right), \end{array}$$

where $B\in R^{3}$ is the magnetic flux density, $m\in R^{3}$ is the magnetic dipole moment of the microrobot, and *∇* is the gradient operator. Further, $\Delta F_{d}\in R^{3}$ represent the inaccuracies in the modeling of the drag forces, $g\in R^{3}$ is the acceleration due to gravity, and V∈R is the volume of the microrobot.

Based on such model, a closed-loop controller is designed to regulate the motion of the microrobots, using the quasi-static electromagnetic fields generated by the testbed (Figure 4). The reference of the controller—provided by the user—is filtered to guarantee continuous derivatives and eliminate components outside of the control bandwidth. The filtered reference is then processed by feed-forward and feed-back controllers.To avoid instabilities and undetermined behavior, the Proportional Integral Derivative (PID) feed-back controller is designed to minimize disturbances with frequencies higher than a decade below that of the tracker [17]. A feed-forward component is added to improve the control performance with an additional control action ($u_{FF}$)

(5) $$u_{FF}=\left[\begin{array}{ccc} x_{d} & {\dot{x}}_{d} & {\ddot{x}}_{d} \end{array}\right]\left[\begin{array}{c} 0\\ -\frac{\rho_{m}C_{D}A}{2}\\ -M \end{array}\right].$$

Finally, the controller outputs forces that are mapped into currents at the electromagnets using a force-current map. As the setup is overactuated, we select a map that aims at minimizing the Frobenius norm of the third-order tensor collecting the Hessian matrices of each component of the field [10,21]. This choice minimizes the spacial variation of the electromagnetic gradient, and consequently, of the electromantic force (3). Overall, such map minimizes the sensitivity of the system to tracking errors.

### 2.6. Vascular Phantom and Drug-Model

Such closed-loop motion control is performed inside an anatomically accurate model of the human vessels [22]. This phantom is fabricated using polydimethylsiloxane (PDMS), due to its tissue-mimicking and optical properties, which allow us to compare the results of ultrasound tracking with those of offline optical tracking [23]. The 20 mm ×20 mm ×20 mm phantom represents a human vessel forking into three different channels. In order to ensure the anatomical accuracy, we construct the phantom so that the sum of the cross-sectional area of the resulting vessels (10 mm${}^{2}$ each) is equal to that of the original vessel (30 mm${}^{2}$). Finally, the phantom is filled with liquid polymerized siloxane to ensure acoustic transparency to ultrasound waves. It is worth noting that, the used polymerized siloxane has a kinematic viscosity of 50 mm${}^{2}$/s, about ten times that of blood [24]. This means that the experiments are conducted in a medium with lower Reynold number than blood. Therefore, interventions in blood would have lower $C_{D}$ (1) and drag forces than those reported in this study.

To further enhance the clinical relevance of the study, the microrobots are coated with a lipid-based thermoresponsive layer. This layer is embedded with a Sudan red dye that allow to identify the behavior of the coating. Moreover, the coating is designed to melt at 39 ${}^{\circ }$C, to minimize the amount of heat necessary for drug-release in the human body.

## 3. Experimental Evaluation

In order to validate the developed setup and techniques, we demonstrate contactless delivery of a drug-model. The intention is to mimic a targeted drug delivery application, in which a microrobot is released by a catheter, steered towards the region of interest where it delivers a drug, and finally returns to the catheter for recollection. Consequently, in the presented experiments, an 800 μm sphere—starting at the end of a channel in the vascular phantom—is steered towards the target area (the end of another channel). As the target is reached, the heating system is activated, triggering the release of the drug-model. After the delivery, the microrobot returns to the starting point where the experiment terminates. In order to guarantee clinical compatibility, the microrobot is tracked using exclusively ultrasound imaging. However, for reasons of comparison and data analysis the procedure is also recorded with a 2D color camera (FLIR Systems, Wilsonville, OR, USA) attached to a microscope (Qioptiq, St Asaph, United Kingdom). Please, refer to the accompanying video for the visualization of the experiment in the Supplementary Materials.

**Figure 5:**
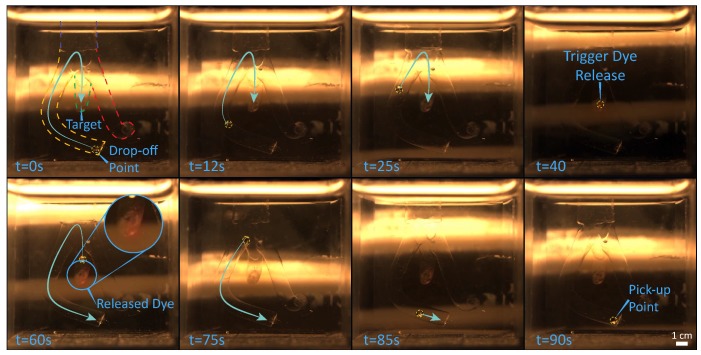
Representative timelapse of the experiments. In the first image, yellow, green, and red dashed lines mark the outline of the channels in the phantom. A yellow dashed line is also used to highlight the position of the microrobot in the frames. The blue arrows approximate the future trajectory of the microrobot. The microrobot starts from the end of the yellow-outlined channel, and navigates to the end of the green one, where it releases the drug-model. Finally, the microrobot returns to the starting point. Moreover, the changes in position of the ultrasound transducer, continuously sweeping through the workspace, can be noticed in the background. Please, refer to the accompanying video for the visualization of the experiment in the Supplementary Materials.

**Figure 6:**
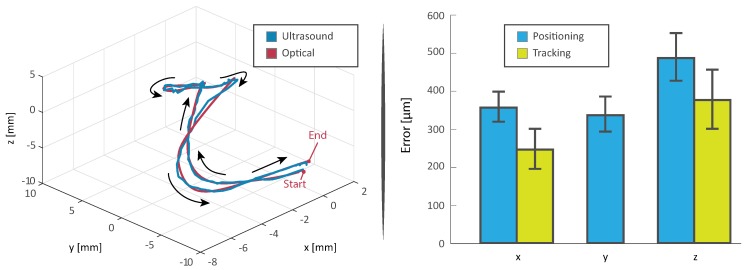
(**Left**) Representative example of the trajectory followed by a microrobot through the experiments. The blue curve shows the ultrasound tracking results. The red curve represent the same trajectory as computed offline by an optical tracker [10]. (**Right**) Histogram of the positioning and tracking errors over the performed ten trials. The positioning error (blue) is defined as the divergence between the filtered reference and the state as tracked by the ultrasound tracking system; i.e., the error as computed in the control loop. Conversely, the tracking error (yellow) is defined as the difference in tracked position between the ultrasound and optical (computed offline) tracker. Consequently, we can only compute the tracking error for *x* and *z* components. However, for reasons of symmetry, we expect the *y* component, which cannot be analyzed optically, to have similar tracking error to that of the *x* component.

The experiment is repeated ten times (Figure 5). The microrobots averagely complete the trials in 212 s, moving with an average velocity of 0.3 mm/s (Figure 6). Moreover, about 20 s are required for the heating process (Figure 1), which is activated as the microrobot is within 10 mm of the designated target. It is worth mentioning that, as the quasi-static and high-frequency magnetic fields can be superimposed without interference, the heating process does not affect the overall completion time. Moreover, this linear superposition, presents other significant advantages; case in point, uninterrupted closed-loop control would be fundamental in the presence of blood flow or dynamic environments.

It can also be noticed that the root mean square value of the positioning and tracking error are 40% and 52% higher for the component normal to the imaging plane (*z*), respectively. This increased error is mainly caused by the diffraction of the ultrasonic wave around the edges of the microrobot. In point of fact, as the diameter of the microrobot is less than ten times the wavelength of the ultrasound, the artifacts resulting from diffraction are comparable in size to the footprint of the microrobot. This phenomenon renders it challenging to determine the exact center of the sphere, as the pixel-count gradient is minimum in the neighborhood of such center (Figure 3). These results, even if somewhat constrained to the tested setup and presenting an additional dimension, are comparable to previous two-dimensional studies regarding motion of microrobots with ultrasound feedback [25].

## 4. Conclusions

This proof-of-concept work presents a biocompatible system for targeted delivery of a drug-model. In order to render the approach fully biocompatible, magnetic fields are used for motion control, a medical ultrasound system is used for imaging, and induction heating is used for active drug release. The presented system is validated in a 3D phantom of human vascular network. In this validation, we simulate a scenario in which the microrobot has been released in a vessel. We navigate such microrobot toward the targeted area, where we trigger the active drug-model release. Finally, we return the robot to the drop-off point, after the release is complete. Compared to previous approaches using induction heating—requiring up to an hour for drug-model release—this approach has an average completion time of 212 s for a release within half a millimeter of the targeted point. In spite of this, a comparison with previous literature using optical feedback shows the motion control performance is clearly constrained by the limited bandwidth of the ultrasound feedback [10,26,27]. Ultrasound scanners with higher refresh-rate that allow higher scanning frequencies could be used to address this issue. Overall, the promising results of this approach, as well as its fast and fully biocompatible nature, render it interesting for further investigation, especially in ex-vivo and in-vivo environments.

Future work will address the limitations of this work to develop a path toward clinical application. For this purpose, we will investigate hardware and smart materials solutions to improve the ultrasound scanning frequency. This will allow to extend the workspace of the quasi-static and high-frequency electromagnetic systems, permitting interventions in the larger parts of the body. Navigation in channels of other size and in the presence of flow will also be investigated. Further, we will test hardware solutions that allow to improve the scanning frequency, therefore increasing the workspace and control bandwidth. Such improvements in hardware and methodology could enable an extensive quantitative analysis with increased number of trials, thereby providing a statistical means to evaluate a myriad of clinically relevant constructs. Finally, we will analyze the use of 3D ultrasound transducers for targeted drug delivery using both individual microrobots as well as swarms. Computer vision and fusion algorithms that allow to address obstructions, such as bones and inhomogeneous tissues, will also be investigated.

## Figures and Tables

**Table 1:** Values of the variables in the model of the microsphere.

Variable	Value	Unit
$\rho_{m}$	9700	[kg/m${}^{3}$]
$C_{D}$	0.5	Pure number
$r_{mr}$	6 × 10${}^{-4}$	[m]
$A_{mr}$	1.131 × 10${}^{-6}$	[m${}^{2}$]
$V_{mr}$	9.0478 × 10${}^{-10}$	[m${}^{3}$]
M	4.1228 × 10${}^{-6}$	[kg]

## Supplementary Materials

The following is available online at https://www.mdpi.com/2072-666X/10/8/504/s1. Video S1: the visualization of the experiment.
